# 

**Published:** 1910-11-19

**Authors:** 


					A NEW BOOK. Crown 8vo. 5s. net.
THE NEWER REMEDIES (a Practical Guide To).
By J. M. Fortescue-Brickdale, M.A., M.D. Oxon., Phys., Clifton
Coll.; Asst. Pliys., B'tol Roy. Infy. ; Lect. Pharm., Univ. of Oxford ; Clin.
Lect., Univ. of Bristol. An account of the Principal New Drugs, with
indications as to their value and employment.
" Will help the harassed practitioner."?Hospital.
" We very cordially recommend it."?South African Medical Record.
Bristol: J. Wright & Sons, Ltd. London : Simpkin & Co., Ltd.
INSTITUTIONAL WORKS.
HOSPITALS AND ASYLUMS OF THE WORLD.
By SIR HENRY BURDETT, K.C.B., K.C.Y.O.
In Four Volumes, with Separate Portfolio containing several hundred Plans.
Vols. I. and II. (ASYLUMS) ... Price ?6 15s. I Vols. III. and IV. (HOSPITALS)
and the Portfolio of Plans ... Price ?9
The Portfolio Of Plans (20 in. X 14 in., drawn to uniform scale), Price ?4 14 6
The Complete Work   ?12 12s.
This magnificent work forms a Complete Survey of the Origin, History, Construction, Administration and Management
of the Hospitals and Asylums of the World, with detailed information concerning the principal Institutions.
Only a few copies remain unsold.
THE SCIENTIFIC PRESS, LTD.,
28 and 29 Southampton Street, Strand, London, W.C.
THE
POCKET-BOOK of TEMPERATURE CHARTS.
Containing seventeen 12-hour and eight 4-hour Charts, perforated so that each Chart may be
easily removed after use.
Bound in paper cover. Suitable size for the pocket.
Price 6<1. net; by post, Tel.
Of all Booksellers, or of
THE SCIENTIFIC PRESS, LIMITED, 28 & 29 Southampton Street, Strand, London, W.C.
Obituary.
The death is announced at Derby of Dr. John Joseph
Bingham, of Alfreton, Derbyshire, at the age of sixty-six.
Dr. Bingham took his diplomas in 1866 and 1867, and
studied at ithe University of 'St. Andrews, -where he
graduated M.D. in 1888. At one time he was house surgeon
at the Derbyshire General Infirmary, and had held the
posts of M.O.H. and public vaccinator at Alfreton District
Belper Union, and M.O., P.O. and Board of Education.
Dr. Charles Otto Gustav Becker has died at Colchester
in his eighty-fourth year. He was born at Minden, served
under Stonewall Jackson in Mexico at the age of fourteen,
and took part in the Schleswig-Holstein campaign, during
which he was cut down in a cavalry charge whilst dressing
the arm of a wounded man, and was taken prisoner by the
Danes. He studied at Gressen where he graduated M.D. in
1853, joining the Army the following year as surgeon. He
also served in the Crimean war, on the staff of Omar
Pasha, and rendered excellent service in the cholera
camp at Yarna. After the Crimean war he became medical
officer at Colchester, where he remained in medical practice
until two years ago.
The death is announced from Paris of Dr. Lancereaux,
at the age of eighty-six. Born in the district of the
Ardennes, he became a member of the Paris Academy of
Medicine in 1877, of which body he subsequently was
elected president. At one time he held the post of
physician to the Paris hospitals. Among his numerous
published works, those on Herpetism, The Intoxications,
and Alcoholism, are the best known, though perhaps the
last brought him the most fame. Though of the old
school, he did not disdain to learn all he could about
recent discoveries in medicine and to discuss them freely.
He was above all a clinician, though he did not deny the
merits of laboratory work. His work as a savant and as
a practitioner was immense. To the end he was remark-
able for his physical and mental vigour, and his simple
unaffected manners endeared him to all with whom he
came into contact. He leaves a widow and family to
mourn his loss.
Dr. Thomas F. S. Caverhill died last week at the resi-
dence of his brother at Jedburgh. He was educated at
Vienna and the University of Edinburgh, graduating M.B..
C.M. at the latter University in 1878, and obtaining the
fellowship of the Royal College of Physicians of Edinburgh
in 1883. He was consulting physician to the Manor Valley
Sanatorium and the author of a work on "The Crusade
against Consumption," and had contributed many articles
on the subject to various medical journals. He was an ex-
president of the Royal Medical Society of Edinburgh, and
had held the posts of resident physician and surgeon at
Edinburgh Royal Infirmary, resident surgeon at the Edin-
burgh Maternity and Simpson Memorial Hospitals, and
physician to the New Town Hospital. He was much in-
terested in the Territorial movement, and had been
surgeon-colonel to the Lothians and Berwick Imperial
Yeomanry.
Appointments and Resignations.
Dr. Henry Jellett, M.D., F.R.C.P.I., King's Professor
of Midwifery, and Gynaecologist to Sir Patrick Dun's Hos-
pital, has been elected Master of the Rotunda Hospital,
Dublin.
W. Stephen Fenwick, M.S., M.B., F.R.C.S., has been
appointed surgeon to out-patients, and W. N. A. Paleyr
M.R.C.S., L.R.C.P., house surgeon at the Gordon Hos-
pital, Vauxhall Bridge Road.
The Mayor of Southampton, Alderman C. J. Sharp, hasr
been appointed chairman of the committee of the South-
ampton Hospital Sunday Fund, in succession to the late'
Mr. Bone. The Fund's Hon. Secretaries have been re-
appointed also in the persons of the Rev. G. S. S. Saunders;
and the Rev. W. Vaughan, assisted by Mr. Gayton.. Mr..
Gayton received at the recent meeting a very warm ac-
knowledgment of his work for the Fund.
244 THE HOSPITAL Nov. 19, 1910
Gifts and Bequests.
Mrs. Rhoda Stuckey Reeves, of Taunton, Somerset,
has left ?200 to the Taunton and Somerset Hc6pifal.
Chelsea Hospital for Women has received a grant of
?105 from the Court of Common Council of the City of
London.
Mr. Alfred Nicholes, of Manor House, Heston,
Middlesex, bequeathed ?100 to the Hounsldw Cottage
Hospital.
Mrs. Mary Walker, of Lea Wood, near Matlock,
Derby, left ?200 to the Derbyshire Royal Infirmary and
jo 100 to the Wirksworth Cottage Hospital.
Miss Burrell, of Botley, Hants, has given ?1,000 to
the Royal Hampshire County Hospital to endow a bed in
memory of her late brother, Mr. R. A. Burrell.
The Saltere* Company contributed twenty guineas to
the Royal Hospital for Incurables, Putney Heath; Mrs.
Bischoffsheim, ?100; Mr. Septimus Yaughan Morgan,
?100; and Sir Charles Cust, ?25.
Mr. George Humphreys, of Carnarvon Road, Redland,
Bristol, one of the oldest members of the Bristol Maeter
Builders' Association, has left ?100 each to the Bristol
General Hospital, the Bristol Royal Infirmary, and the
Bristol Hospital for Sick Women and Children.
Mr. Frederick Henry Harrison, of White Hall,
Lincoln, chairman of Messrs. Harrison and Co., Limited,
Iron and Steel Works, has left on the death of his wife
?1,000 to the Boston Hospital, ?250 to the Lincoln County
Hospital, ?200 to the Medical Missions, and ?100 to the
Lincoln General Dispensary.
We are glad to note that University College Hospital
has received the following new annual subscriptions :
E. Schumacher, Esq., ?3 3s. ; the Most Hon. the Marquis
Camden, ?2 2s.; Herbert Flemming, Esq., per P. Flem-
ming, Esq., F.R.C.S., ?2 2s. ; Miss B. Mackinnon, ?2 2s. ;
H. Kcenisberg, Esq., ?2 2s.; Messrs. Ewart and Son,
Limited, ?2 2s.
Prince Alexander of Teck has received a donation of
?100 from Queen Alexandra for the Prince Francis of
Teck Memorial Fund, with her Majesty's best wishes
for the success of the appeal and the prosperity of the
hospital; the following donations have also been received :
Anonymous, ?1,000; Mr. V. W. Wallace, ?50; and Sir
Maurice and Lady FitzGerald, ?25.
Jottings.
The fifty-sixth annual meeting of the Royal Hospital for
Incurables, Putney Heath, will be held at the Cannon
Street Hotel on Friday, November 25, at 11.30 a.m.
The Right Hon. the Earl of Shaftesbury, K.C.V.O., will
preside at a dinner in support of a special appeal for
?8,500 to free the Queen's Hospital for Children from
debt on Wednesday, November 23, at the Hotel Cecil,
7.30 for 8 r.M.
The fifty-fifth annual festival dinner of the Poplar
Hospital for Accidents, Poplar E., will be held at the
Holborn Restaurant, High Holborn, on Wednesday,
December 14, 1910, at 7 p.m. precisely. Sir Owen
Philipps, K.C.M.G., M.P., will preside.
Mrs. William Adamson will open the new out-patient
department of the Royal Southern Hospital, Liverpool,
on Monday, November 21, at 3 p.m. Mrs. W. H. Lever
will afterwards open the " Hulme" Lift, presented by
her husband. The Lord Mayor-Elect, S. Mason Hutchin-
son, Esq., has kindly consented to preside.
A festival dinner in aid of the City of London Hospital
for Diseases of the Chest was held at the Trocadero
Restaurant last week. The Hon. Walter Rothschild
(vice-president), who occupied the chair, said that the
institution was in a state of grave need. It was
necessary to raise ?3,0C0 before the end of the year. The
secretary announced contributions amounting to ?2,617,
including 200 guineas from the Bethnal Green Working
Men's Society.
The Duchess of Albany presided at a meeting of the
Jubilee Committee of the National Hospital for Paralysed
and Epileptics at Queen Square, Bloomsbury, cn Monday
last, and explained the steps taken to raise ?50,000 to
complete the building of the institution, and expressed
her gratitude for the help received. She recalled King
Edward's visit to the hospital a year ago, when he ex-
pressed his sympathy with their labours and the deepest
grief that so generous and distinguished a patron as tlw
Duke of Albany should have been called away. The
Duchess added that ?26,000 had already been raised.
TO HOSPITAL SECRETARIES AND OTHERS.
We invite contributions from any of our readers, and
shall be glad to pay, according to length, for items of news
for the institutional section (including appointments, resig-
nations, gifts, etc.) which we may publish. All payments
for manuscripts used are made as early as possible after
the beginning of each quarter.
GENERAL PRACTITIONERS'
CONTRIBUTIONS.
The Hospital devotes a special page to General
Practitioners' contributions. We therefore invite
from practitioners contributions based upon their
experience in the management of cases, and in the
treatment^and diagnosis of disease; especially shall
we be prepared to welcome articles dealing, practi-
cally, with treatment, and with the use and value
of new remedies and methods.
No article should exceed 1,100 words, and, if
accepted, one guinea for a contribution of this
length will be paid to the writer after publication.
Each communication should be accompanied by a
stamped directed envelope for the return of the MS.
if found unsuitable.
Contributions should be written, or preferably
typed, on one side of the paper only, and all articles
sent in are accepted upon the distinct understanding
that they are forwarded to The Hospital only.
BUSINESS NOTICES.
Annual Subscriptions.
The rate of annual subscriptions to The
Hospital, either direct from the Office or through
agents, is: ?
For the United Kingdom  15s. a year.
For the Colonies and Abroad ... 17s. ,,
For the United Kingdom (with
Insurance Policy)  ?1 ,,
inclusive of postage in each case.
Letters relating to the Publishing, Sale and
Advertisement Departments must be addressed to
the Manager (not to the Editor): ?
The Manager,
The Hospital Building,
28 and 29 Southampton Street,
Strand, London, W.C.
THE IDEAL WORK OF REFERENCE.
BURDEN'S HOSPITALS & CHARITIES
1910 EDITION.
Zhe JlJeaiVBook of philanthropy anfc Ibospital annual.
Edited by SIR HENRY BURDETT, K.C.B., K.C.V.O.
A Complete Guide and Directory of the Hospitals, Asylums, and Charitable Institutions
throughout the English-speaking World.
Contains Special New Chaptebs on :?
Voluntary Hospitals in 1909.?Soundness of the Voluntary System.?The need of greater zeal on the
part of those who accept office.?State-aided Hospitals in the U.S.A. and Canada.?The Appropriation
of Public Funds for the partial Support of Voluntary Hospitals in U.S.A. and Canada.?The Need of an
Apostle and of greater interest on the part of the Clergy, &c., &c.
TWENTY-FIRST YEAR OF PUBLICATION.
Over 1,000 pages. Price 10/6 net; Post Free, 10/11.
ORDER your copy of the Medical Whitaker NO'W.
Publishebs :
THE SCIENTIFIC PRESS, LIMITED, 28 & 29 Southampton Street, Strand, London, W.C.

				

